# Ferritin levels in children and adolescents with type 1 diabetes mellitus: relationship with microvascular complications and glycemic control

**DOI:** 10.20945/2359-3997000000279

**Published:** 2020-08-24

**Authors:** Kotb Abbass Metwalley, Duaa Mohamed Raafat, Deiaaeldin Mohammed Tamer, Hekma Saad Farghaly, Ghada Mohamed Said

**Affiliations:** 1 Assiut University Faculty of Medicine Department of Pediatrics Assiut Egypt Department of Pediatrics, Faculty of Medicine, Assiut University, Assiut, Egypt; 2 Assiut University Faculty of Medicine Department of Clinical Pathology Assiut Egypt Department of Clinical Pathology, Faculty of Medicine, Assiut University, Assiut, Egypt

**Keywords:** Ferritin, microvascular complications, glycemic control, microalbuminuria, diabetes mellitus

## Abstract

**Objective:**

Evaluate ferritin levels in children and adolescents with type 1 diabetes mellitus and its relation to diabetic microvascular complications, and metabolic control.

**Subjects and methods:**

This study included 180 children and adolescents with type 1 diabetes mellitus (T1DM) with a mean age of 14.9 ± 3.1 years and 180 apparently normal children matched for age and sex (control group). All children were evaluated with full history taking, thorough clinical examination, laboratory assessment of high-sensitivity C-reactive protein and hemoglobin A1c (HbA1c), and evaluation of the presence of microvascular complications. Serum ferritin levels were measured using electrochemiluminescence immunoassay. The patients were divided into two groups according to the presence or absence of microvascular complications.

**Results:**

Serum ferritin levels were significantly higher in patients with T1DM in both groups compared with healthy controls (p < 0.001). Additionally, patients with microvascular complications had higher serum ferritin concentrations than those without microvascular complications (p < 0.001). Patients with microalbuminuria showed higher ferritin levels compared with patients without microalbuminuria (p < 0.05). Stepwise regression analysis revealed that levels of HbA1c and urinary albumin excretion were independently related to ferritin levels (p < 0.001 for both). On receiver operating characteristic (ROC) curve analysis, a ferritin cutoff value of 163.6 ng/mL differentiated patients with microvascular complications from those without microvascular complications with a sensitivity of 92.1% and specificity of 93.4%.

**Conclusion:**

Serum ferritin levels are elevated in T1DM, particularly in patients with microvascular complications.

## INTRODUCTION

Diabetes is a group of metabolic diseases characterized by chronic hyperglycemia associated with long-term damage, dysfunction, and failure of various organs ( 1 ). Prevention of long-term chronic complications has now become one of the main goals in modern treatment for type 1 diabetes mellitus (T1DM) in children ( 2 ). Beyond impaired insulin secretion and resistance, inflammation has attracted much attention as a contributor to the development of diabetes and its long-term complications ( 3 ). Ferritin is generally used as an index of the extent of iron storage in the body but is also used as an acute phase reactant in response to inflammation ( 4 ). It is unclear whether increased ferritin reflects or causes inflammation, or if it is involved in the inflammatory cycle ( 5 ). Still, serum ferritin levels have only rarely been investigated in children and adolescents with T1DM ( 6 ). Therefore, the aim of this study was to evaluate serum ferritin levels in a cohort of children and adolescents with T1DM and the relation of ferritin levels with diabetic microvascular complications and metabolic control.

## SUBJECTS AND METHODS

### Patients

This study included 180 children and adolescents with T1DM defined according to the criteria of the American Diabetes Association ( 7 ) and ages ranging between 12 and 18 years (mean 14.9 ± 3.1 years). The patients were recruited over a period of 1 year from the outpatient pediatric diabetes clinic at Children's Hospital, Assiut University (Assiut, Egypt). The study also included 180 healthy children recruited from the general population and matched for age, sex, and socioeconomic status. Excluded from the study were patients who underwent surgery within the previous 2 months or had intercurrent illnesses, connective tissue disease, other autoimmune disorders, malignancy, or anemia. Patients on antiplatelet drugs, lipid-lowering medications, or antihypertensive therapies other than captopril were also excluded. Captopril was only given for patients with microalbuminuria as a treatment for nephropathy. The patients were stratified into two groups according to the presence of microvascular complications. Group 1 included 68 patients with microvascular complications from TIDM; these patients had either one or more of the following inclusion criteria: diabetic kidney disease, retinopathy, or neuropathy. Group 2 (without microvascular complications) included 112 children with T1DM without the above-mentioned microvascular complications both during the study and in their previous records.

## METHODS

The medical history of all participants was evaluated, including age at T1DM onset, diabetes duration, glycemic control, and type and dose of insulin. The clinical examination included blood pressure (BP), height, and weight measurements, body mass index (BMI) calculation, and neurological and fundus examination. BP was measured using a conventional sphygmomanometer with the child in the seated position after 5 minutes of rest. The size of the sphygmomanometer cuff for BP measurement was chosen according to the child's age and size. If the child was above the 90th percentile for age and sex, BP levels were measured twice for the validity of the reading. Standard deviation (SD) scores (SDSs) for the mean BP were calculated according to the report of the National High Blood Pressure Education Program Working Group on High Blood Pressure in Children and Adolescents ( 8 ). Hypertension was defined as a mean systolic or diastolic BP > 1.645 SDSs, in which the 1.645 SDS value corresponds to the 95th percentile in a standard normal distribution. Height and weight were measured using a wall-mounted stadiometer and a calibrated weight scale with the child wearing only underwear. BMI was calculated using the formula BMI = weight (kg)/height (m^2^) and was expressed in SDSs based on Egyptian growth reference data ( 9 ). Full neurological examination was performed in patients without known diabetic neuropathy to detect evidence of peripheral neuropathy ( 10 ). Clinical manifestations of peripheral neuropathy include paresthesias, neuropathic pain, abnormal 10-g Semmes-Weinstein monofilament test, decreased vibration sense, diminished or lost ankle reflexes, and motor weakness, confirmed by a nerve conduction velocity study ( 11 ). Diabetic retinopathy was evaluated by retinography and fluorescein angiography. The patients were classified as having diabetic retinopathy if the following diabetic retinal changes were present: vitreous hemorrhage, macular traction detachment, phthisis or enucleation secondary to proliferative diabetic retinopathy, or macular edema ( 12 ). The occurrence of diabetic kidney disease was evaluated by urinary albumin excretion ( 13 ).

The study protocol was approved by the Ethics Committee of the Faculty of Medicine at Assiut Children University Hospital (Assiut, Egypt).

### Laboratory investigation

Venous blood samples were collected in the morning after 10 to 12 hours of overnight fasting. Concentrations of serum total cholesterol, high-density lipoprotein (HDL) cholesterol, and triglycerides were measured by standard enzymatic methods and reagents (Boehringer Mannheim GmbH, Penzberg, Germany) with a fully automated analyzer. Concentrations of low-density lipoprotein (LDL) cholesterol were calculated using the Friedewald's equation ( 14 ). High-performance liquid chromatography (Variant Analyzer; Bio-Rad Laboratories, Inc., Marnes-la-Coquette, France) was used to measure hemoglobin A1c (HbA1c) levels. Levels of high-sensitivity C-reactive protein (hs-CRP) were measured using the High Sensitivity C-Reactive Protein (hs-CRP) Enzyme Immunoassay Test (ELISA) kit for quantitative determination of C-reactive protein concentrations in human serum (catalog no. E29-056; Immunospec Corp., Canoga Park, CA, USA). Serum ferritin levels were determined using electrochemiluminescence immunoassay (Modular Analytics E170; Roche Diagnostics, Rotkreuz, Switzerland). Urinary albumin excretion (as an indicator of nephropathy) was measured in early morning urine samples as albumin-to-creatinine ratio by an immunonephelometric method on a prime photometer (BPC BioSed, Rome, Italy). Microalbuminuria and macroalbuminuria were considered to be present if urinary albumin excretion in at least two out of three consecutive urine samples collected 2 months apart was 30-299 mg/g creatinine and ≥300 mg/g creatinine, respectively ( 15 ). Potential factors affecting urinary albumin excretion, such as exercise, fever, and posture, were excluded ( 16 ).

### Statistical analysis

The statistical analysis was carried out using SPSS software, version 16 (IBM Corp, Armonk, NY, USA). Quantitative variables were described as mean and SD values, while qualitative variables were described as numbers and percentages. Student's *t* test was used as a significance test to compare ferritin levels in patients with microvascular complications versus those without microvascular complications and in patients with microvascular complications versus healthy controls. Correlation studies were performed using Pearson's correlation coefficient. Multiple regression analysis was employed to assess the relation between ferritin and clinical and laboratory variables. Logistic regression was used to examine the relation between ferritin and microvascular complications after adjustment for other variables. Receiver operating characteristic (ROC) curve was used to determine the best ferritin cutoff value to detect microvascular complications. A p value < 0.05 was considered significant in all analyses.

## RESULTS

The most common chronic complication encountered in the study patients with T1DM was microalbuminuria, followed by peripheral neuropathy and retinopathy ( Table 1 ). Serum ferritin levels were significantly higher in patients with T1DM (n = 180) compared with healthy controls (n = 180; 502.35 ± 121.17 *vs.* 80.76 ± 54.13, respectively; p < 0.001). Patients with and without microvascular complications, when compared with control subjects, had significantly higher systolic and diastolic BP SDSs, along with significantly higher urinary albumin creatinine ratio and serum levels of total cholesterol, LDL cholesterol, triglycerides, HbA1c, and hs-CRP and lower serum HDL cholesterol levels. Patients with microvascular complications were older and had longer disease duration. They also had significantly higher levels of systolic and diastolic BP, urinary albumin excretion, serum HbA1c, hs-CRP, and lipids (except for HDL cholesterol), and used a higher insulin dose compared with those without microvascular complications. Ferritin levels were also significantly greater in both diabetic groups when each group was compared separately with healthy control subjects (p<0.001). Notably, patients with microvascular complications had significantly higher serum ferritin concentrations at the time of evaluation than patients without microvascular complications (p<0.001). The difference between the two groups as regards ferritin remained significant after adjustments for age and disease duration (p < 0.001) ( Table 2 ). The ROC curve analysis revealed that a ferritin cutoff value of 163.6 ng/mL differentiated patients with microvascular complications from those without microvascular complications with a sensitivity of 92.1% and specificity of 93.4% (area under the curve 0.92, 95% confidence interval [CI] 0.91-1.10, p < 0.001). The analysis of the patients’ clinicopathological characteristics in relation to ferritin levels revealed that patients with microalbuminuria had significantly higher ferritin levels compared with those without microalbuminuria (607.46 ± 224.94 *vs.* 256.76 ± 88.19, respectively; p < 0.001). Moreover, patients with peripheral neuropathy or diabetic retinopathy had higher ferritin levels than those without these complications, although the difference did not reach statistical significance (p > 0.05). Significant positive correlations were observed between levels of ferritin with age (p < 0.0001), disease duration (p < 0.001), systolic BP SDS (p < 0.01), diastolic BP SDS (p < 0.05), HbA1c (p < 0.001), and urinary albumin excretion (p < 0.05), while no correlation was found between ferritin and serum lipids, hs-CRP, BMI SDS, or insulin requirements (p > 0.05) ( Table 3 ). On stepwise regression analysis, HbA1c and urinary albumin excretion were independently related to ferritin levels (p < 0.001) ( Table 4 ). Moreover, logistic regression revealed that ferritin was a significant independent factor for microvascular complications after adjustment for other variables, namely, age, gender, disease duration, BMI, blood pressure, HbA1c, urinary albumin excretion, and fasting lipids (odds ratio 3.15, 95% CI 2.26–5.44, p < 0.001).

**Table 1:** Clinical and laboratory characteristics of the patients with diabetes

Variable		Patients (n = 180)
Age (years)		14.9 ± 3.1
M/F		108/72
Diabetes duration (years)		8.6 ± 2.8
BMI SDS (mean ± SD)		0.65 ± 0.3
	Systolic BP SDS (mean ± SD)	1.35 ± 0.4
Diastolic BP SDS (mean ± SD)		0.45 ± 0.15
Triglycerides (mg/dL)		170.7 ± 18.6
Total cholesterol (mg/dL)		183.7 ± 13.6
LDL cholesterol (mg/dL)		112.7 ± 33.5
HDL cholesterol (mg/dL)		46.7 ± 16.9
UACR (mg/g creatinine)		188.2 ± 34.8
Hb1Ac (%; mean ± SD)		9.2 ± 3.5
hs-CRP (mg/L)		4.9 ± 0.94
Ferritin (ng/mL)		502.35 ± 121.17
Microalbuminuria, n (%)		36 (20%)
Retinopathy, n (%)		12 (6.6%)
Peripheral neuropathy, n (%)		24 (13.3%)

Data are shown as mean ± standard deviation values, unless otherwise specified.

M: male; F: female; BMI: body mass index; SDS: standard deviation score; SD: standard deviation; BP: blood pressure; HDL: high-density lipoprotein cholesterol; UACR: urinary albumin creatinine ratio; HbA1c: hemoglobin A1c; hs-CRP: high-sensitivity C-reactive protein; LDL: low-density lipoprotein cholesterol.

**Table 2:** Clinical and laboratory variables of patients with type 1 diabetes and healthy controls

	Patients with microvascular complication (n = 68) (Group 1)	Patients without microvascular complication (n = 112) (Group 2)	Healthy controls (n = 180)
Age	16.4 ± 2.6 * , #	13.7 ± 3.6	14.1 ± 3.2
Sex M/F	40/28 *	76/36 *	102/78
Diabetes duration (years)	10.2 ± 2.3 #	7.1 ± 1.1	−
Insulin dose (U/kg per 24 h)	1.5 ± 0.3 #	0.9 ± 0.1	−
BMI SDS	0.66 ± 0.7	0.63 ± 0.3	0.62 ± 0.5
Systolic BP SDS	1.7 ± 0.3*, #	1.3 ± 0.2 *	0.64 ± 0.3
Diastolic BP SDS	0.98 ± 0.3*, #	0.4 ± 0.2 *	0.31 ± 0.04
Triglycerides (mg/dL)	181.9 ± 34.8*, #	124.7 ± 19.9 *	103.1 ± 10.2
Total cholesterol (mg/dL)	199.6 ± 34.2*, #	165.2 ± 29.1 *	126.3 ± 17.3
LDL cholesterol (mg/dL)	138 ± 33.6*, #	99.7 ± 23.7 *	86.7 ± 12.2
HDL cholesterol (mg/dL)	41.0 ± 15.0*, #	61.7 ± 15.9 *	69.8 ± 27.9
UACR (μg/mg creatinine)	205.6 ± 35.6 #	80.2 ± 22.9	−
Mean Hb1Ac (%)	9.1 ± 1.1*, #	7.5 ± 0.5 *	4.8 ± 0.2
hs-CRP (mg/L)	6.02 ± 1.3*, #	2.81 ± 0.73 *	0.38 ± 0.06
Ferritin (ng/mL)	687.54 ± 212.14*, #	398.76 ± 185.48 *	80.76 ± 54.13

Data are shown as mean ± standard deviation, unless otherwise specified.

* Significance versus control subjects.

# Group with microvascular complications *vs.* group without microvascular complications.

M: male; F: female; BMI: body mass index; SDS: standard deviation score; BP: blood pressure; LDL: low-density lipoprotein; HDL: high-density lipoprotein; UACR: urinary albumin creatinine ratio; HbA1c: hemoglobin A1c; hs-CRP: high-sensitivity C-reactive protein.

**Table 3:** Correlations between ferritin levels and demographic, clinical, and laboratory variables

Variable	r	P value
Age (years)	0.70	0.001
Disease duration (years)	0.876	0.001
Systolic BP SDS	0.347	0.05
Diastolic BP SDS	0.361	0.05
HbA1c (%)	0.765	0.001
UACR (mg/g creatinine)	0.554	0.01

BP: blood pressure; HbA1c: hemoglobin A1c; SDS: standard deviation score; UACR: urinary albumin creatinine ratio.

**Table 4:** Multiple regression analysis of the relation of ferritin to clinical and laboratory variables

Variable	Standardized coefficients	P value
Age (years)	0.19	0.36
Disease duration (years)	0.27	0.08
Systolic BP SDS	0.18	0.48
Diastolic BP SDS	0.03	0.91
HbA1c (%)	0.65	<0.001
UACR (mg/g creatinine)	0.44	<0.001

BP: blood pressure; HbA1c: hemoglobin A1c; SDS: standard deviation score; UACR: urinary albumin creatinine ratio.

## DISCUSSION

In this study, serum ferritin levels were significantly higher in patients with T1DM compared with healthy individuals. The increase in serum ferritin concentrations was more evident in patients with microvascular complications than in those without these complications or in healthy controls. Also, ferritin levels were found to influence microvascular complications independently of other risk factors. Similar results have also been reported in patients with type 2 diabetes, suggesting that increased ferritin is an important finding in diabetes mellitus irrespective of the disease type ( 17 ). Indeed, Borah cols. ( 18 ) have reported that type 2 diabetes mellitus is associated with increased serum ferritin, and that this finding may have a major role in the development of diabetes and its complications. Moreover, among patients without diabetes, increased ferritin levels were independently associated with the development of metabolic syndrome ( 19 ). Padwal cols. ( 20 ) found increased levels of ferritin in nondiabetics with prediabetes and metabolic syndrome, suggesting that patients with metabolic syndrome have a proinflammatory state independent of glucose tolerance status. In contrast, Carenini cols. ( 6 ) reported that diabetes *per se* did not influence the serum concentrations of ferritin among 30 male patients with T1DM and aged between 15 and 64 years. These conflicting results could be explained by differences in age, number of cases, and methodology of each study, along with a founder effect (genetic factors) ( 21 ). Ferritin can cause cell damage through mechanisms involved in increased oxidative stress, activation of inflammatory cytokines, and infiltration of macrophages ( 22 ). Excessive iron deposits produce hydroxyl radicals, which cause lipid peroxidation leading to DNA fragmentation and tissue damage ( 23 ). These harmful effects, which have been demonstrated in experimental animal models, can be ameliorated by reducing iron levels ( 24 ).

We found in the present study significant positive correlations between levels of ferritin and HbA1c (p < 0.001), while stepwise regression analysis revealed that HbA1c levels were independently related to ferritin levels. This may explain why poor metabolic control may play a role in the proinflammatory state found in patients with T1DM ( 25 ). Some studies link poor glycemic control to increased inflammation and atherothrombotic risk in patients with diabetes mellitus and have shown that improved metabolic control is associated with a significant reduction in ferritin levels ( 26 , 27 ). The vicious cycle consists of high HbA1c levels, hyperglycemia, glycation of hemoglobin releasing free iron, and ferritin enhancing the generation of oxidants and tissue damage ( 28 ).

In the present study, patients with microalbuminuria had significantly higher levels of ferritin compared with those without microalbuminuria (p < 0.001). Significant positive correlations were found between ferritin and urinary albumin excretion (p < 0.01), and stepwise regression analysis revealed that urinary albumin excretion was independently related to ferritin levels. Hsu and cols. ( 29 ) have reported that ferritin may cause microalbuminuria in patients of type 2 diabetes mellitus. Inflammatory activity of ferritin leads to increased vascular permeability, thickening of the glomerular basement membrane, and endothelial cell apoptosis, which contribute to the development of nephropathy ( 30 ).

To our knowledge, this is the first report to define a cutoff value (163.6 ng/mL) for ferritin level indicating the presence of microvascular complications, which can be anticipated at the time of diagnosis with a sensitivity of 92.1% and specificity of 93.4%. This suggests that ferritin has potential clinical usefulness in T1DM and may be considered an additional suitable, inexpensive, and adequate prognostic marker for reliably detecting microvascular complications in children and adolescents with T1DM. This may lead to improved management and, consequently, prognosis of patients with T1DM. Still, prospective studies are needed to verify these results, and the ferritin cutoff value to detect complications should be further validated in another group of patients with T1DM.

The present study has some limitations, including the small sample size and the fact that the results were obtained from a single hospital, therefore having a high probability of selection bias. Also, the patients with increased urinary albumin excretion were on therapy with captopril, which has antiinflammatory properties that may down-regulate ferritin levels.

In conclusion, serum ferritin levels are elevated in patients with T1DM, particularly in those with microvascular complications, and are significantly correlated with glycemic control. Therefore, measurement of serum ferritin levels in poorly controlled patients might help identify those at high risk of developing microvascular complications.
